# Resilience of Biocontrol for Aflatoxin Minimization Strategies: Climate Change Abiotic Factors May Affect Control in Non-GM and GM-Maize Cultivars

**DOI:** 10.3389/fmicb.2019.02525

**Published:** 2019-11-08

**Authors:** Alessandra Marcon Gasperini, Alicia Rodriguez-Sixtos, Carol Verheecke-Vaessen, Esther Garcia-Cela, Angel Medina, Naresh Magan

**Affiliations:** Applied Mycology Group, Cranfield Soil and Agrifood Institute, Environment and Agrifood Theme, Cranfield University, Bedford, United Kingdom

**Keywords:** resilience, biocontrol, aflatoxins, climate change, non-toxigenic *Aspergillus flavus*, non-GM maize, GM maize

## Abstract

There has been significant interest in the development of formulations of non-toxigenic strains of *Aspergillus flavus* for control of toxigenic strains to reduce the aflatoxin B_1_ (AFB_1_) contamination of maize. In the future, climate change (CC) abiotic conditions of temperature (+2–4°C), CO_2_ (existing levels of 400 vs. 800–1,200 ppb), and drought stress will impact on the agronomy and control of pests and diseases. This study has examined (1) the effect of two-way interacting factors of water activity × temperature on colonization and AFB_1_ contamination of maize cobs of different ripening ages; (2) the effect of non-toxigenic strains of *A. flavus* (50:50 inoculum ratio) on relative control of toxigenic *A. flavus* and AFB_1_ contamination of ripening cobs; (3) post-harvest control of AFB_1_ by non-toxigenic strains of *A. flavus* in non-GM and isogenic GM maize cultivars using the same inoculum ratio; and (4) the impact of three-way interacting CC factors on relative control of AFB_1_ in maize cobs pre-harvest and in stored non-GM/GM cultivars. Pre-harvest colonization and AFB_1_ production by a toxigenic *A. flavus* strain was conserved at 37°C when compared with 30°C, at the three ripening stages of cob development examined: milk ripe (R3), dough (R4), and dent (R5). However, pre-harvest biocontrol with a non-toxigenic strain was only effective at the R3 and R4 stages and not at the R5 stage. This was supported by relative expression of the *aflR* regulatory biosynthetic gene in the different treatments. When exposed to three-way interacting CC factors for control of AFB_1_ pre-harvest, the non-toxigenic *A. flavus* strain was effective at R3 and £4 stages but not at the R5 stage. Post-harvest storage of non-GM and GM cultivars showed that control was achievable at 30°C, with slightly better control in GM-cultivars in terms of the overall inhibition of AFB_1_ production. However, in stored maize, the non-toxigenic strains of *A. flavus* had conserved biocontrol of AFB_1_ contamination, especially in the GM-maize cultivars under three-way interacting CC conditions (37°C × 1,000 ppm CO_2_ and drought stress). This was supported by the relative expression of the *aflR* gene in these treatments. This study suggests that the choice of the biocontrol strains, for pre- or post-harvest control, needs to take into account their resilience in CC-related abiotic conditions to ensure that control of AFB_1_ contamination can be conserved.

## Introduction

There has been significant interest in developing biocontrol agents for aflatoxin B_1_ (AFB_1_) control in staple commodities, especially maize. Indeed, there are some commercial products based on individual non-aflatoxigenic *A. flavus* strains or mixtures of such strains for reducing AFB_1_ contamination of maize and groundnuts in West and East Africa and in the USA for control of toxin contamination in cotton and groundnuts (Lyn et al., 2009; Abbas et al., 2011; Bandyopadhyay et al., 2016; Mauro et al., 2018; Kagot et al., 2019).

Abiotic factors, especially drought stress, can have a major impact on maize growth especially during the critical silking period, which can allow both pest damage and increase in the colonization by *A. flavus* resulting in AFB_1_ contamination. In some cases, pest damage has been reduced by the use of GM cultivars, which have resistant genes for pesticides and/or herbicides. This can reduce the entry points for *A. flavus* and reduce contamination. However, under expected climate change (CC) conditions, which involves interactions between key abiotic factors such as the predicted increase in environmental CO_2_ (400 vs. 1,000–1,200 ppm), elevated temperature (+2–4°C), and extreme changes in drought/wet regimes, there have been few studies to examine the resilience of non-toxigenic *A. flavus* biocontrol strains used for toxin reduction in maize and groundnuts. Many maize growing regions are considered hot spots for an impact of these environmental pressures. Indeed, work on *A. flavus* colonization of maize grain has shown that interacting conditions of +2–5°C, elevated CO_2_ (650–1,000 ppm), and drought stress can result in an increase in both regulatory (*aflR*) and structural genes (*aflD*) involved in AFB_1_ biosynthesis as well as other secondary metabolite genes in maize grain and lead to a significant stimulation in AFB_1_ contamination (Battilani et al., 2016; Medina et al., 2017a; Gilbert et al., 2018). Bearing this in mind, it is thus surprising that while biocontrol of toxigenic *A. flavus* using microbial antagonists and non-toxigenic strains of *A. flavus* has been examined for many years, their resilience has never been examined under expected CC regimes (Cotty, 1994; Abbas et al., 2011; Bandyopadhyay et al., 2016; Weaver et al., 2016; Kagot et al., 2019). Some studies have examined the impact of non-toxigenic *A. flavus* strains and other microbial biocontrol agents on temporal control of AFB_1_ in maize stored under different temperature and water availabilities (Mohale et al., 2013; Al-Saad et al., 2016; Medina et al., 2017a). However, the relative resilience of the biocontrol strains for control of mycotoxin production under CC scenarios has not previously received any attention (Medina et al., 2017b). It is very important to understand how the potential biocontrol strains targeting AFB_1_ control in maize may behave under interacting CC abiotic factors and whether they have the necessary resilience to reduce biosynthesis of AFB_1_ *in situ*.

The objectives of this study were to examine (1) the effect of ripening stage of maize cobs on rates of colonization and AFB_1_ production by *A. flavus* in relation to interactions between two-way abiotic factors of temperature × water availability (water activity, a_w_); (2) the effect of these two-way interacting factors on the control of AFB_1_ contamination using 50:50 ratios of non-toxigenic and toxigenic strains in maize cobs of different ripening stages and in stored non-GM and isogenic GM maize cultivars; and (3) the effect of three-way interacting CC abiotic factors on resilience of non-toxigenic *A. flavus* strains in terms of reducing the expression of key biosynthetic genes involved in aflatoxin synthesis, and on phenotypic AFB_1_ contamination, in these two types of maize cultivars.

## Materials and Methods

### Fungal Strains

Two non-toxigenic strains of *A. flavus* isolated from Mexican and Brazilian maize were used in these experiments (AFL^−^Mex02; AFL4^−^). Both strains were examined molecularly to confirm that key genes in the biosynthetic cluster for aflatoxins were deleted using the multiplex PCR method developed by Callicott and Cotty (2015). Different toxigenic strains including one Mexican strain (ALF^+^-Mex01, isolated from Mexican maize), a type strain NRRL3375 (AFLe^+^), and one Brazilian toxigenic strain (AFLb^+^, isolated from Brazilian maize), all known AFB_1_ producers were used. Table 1 summarizes the strains used in this study. The type strain was kindly provided by Prof. D. Bhatnagar, Southern Regional Research Centre, New Orleans, LA, USA.

**Table 1:** List of toxigenic and non-toxigenic strains used in this study.

Strain code	Source	Aflatoxin B_1_ producer +/−	Deleted genes
AFL-MEX01^−^	White maize/Mexico	Naturally very low producer^*^	None
AFL-MEX02	White maize/Mexico	Producer	
AFL4^−^	GM maize P30F53 H®, Brazil	Non-producer	*aflN, aflE, aflS*. *aflB. aflA*
AFLB^+^	Landrace maize red grain, Brazil	Producer	
NRRL3357	Peanuts (type strain, ARS, USDA)	Producer	

* *Less than the limit of quantification*.

### Pre-harvest Studies With Maize Cobs of Different Ripening Ages for Resilience of Biocontrol of AFB_1_

Maize cobs of different ripening stages (R3: Milk; R4: Dough; R5: Dent) were obtained from the NIAB farm (National Institute of Agriculture and Botany; Cambridge, UK). The type of maize was ES Regain (Euralis Semences; forage maize). Harvested maize cobs of different ripening ages were brought to the laboratory where the water activity (a_w_) of sub-samples of detached kernels from the entire cob (5–10 maize kernels from the apical, middle, and distal parts of the cobs) was measured (AQUALAB® Series TE4; Decagon Devices Inc., Pullman, WA, USA). Maize cobs were then divided into batches and snap frozen in liquid nitrogen and stored at −20°C for later use in the experiments. The a_w_ of the R3 (milk ripe), R4 (dough), and R5 (dent) stages was found to be 0.985, 0.976, and 0.958, respectively.

The fungal strains were all point inoculated on 3% Maize Meal Agar (MMA, 0.98 a_w_) and incubated at 25°C for 7 days, and the conidial spore suspensions made by using a loop and decanting spores into 9 ml of sterile water +0.5% Tween 8-0 solution in 25 ml Universal bottle. The conidial spore concentrations were measured with a hemocytometer, and then diluted with sterile water as required, to obtain a final concentration of 1 × 10^4^ spores/ml.

The flash-frozen cobs were thawed at 4°C for 24 h. The maize cobs were divided into three segments, and these were point inoculated. Each treatment consisted of 3–4 replicate maize cob segments taken at random from the batch of each ripening age. These were point inoculated by damaging a single kernel with a surface sterilized needle and then decanting a 10 μl droplet containing 10^4^ conidia/ml of the *A. flavus* type strain. These were incubation in separate environmental chambers at 30 and 37°C. The equilibrium relative humidity (ERH) of the atmosphere was maintained at the actual a_w_ levels of the cobs by using glycerol/water solutions of the same a_w_ as the maize cob ripening stages. The colonization rate was measured over a 10-day period, and the AFB_1_ contamination quantified at the end of the experimental period only.

Subsequent studies examined the biocontrol of the toxigenic *A. flavus* strain and AFB_1_ control in each of the ripening stages as described previously by Samsudin et al. (2017). The different ripening stages of the cobs were incubated in separate environmental chambers to stabilize for 3 hours at 25°C until inoculation. The control treatment used was inoculated with the toxigenic *A. flavus* (MEX01^+^) or the type NRRL3375 strain alone. The biological control agent (BCA) treatments consisted of a 50:50 conidial inoculum ratio of pathogen:antagonist. This was used based on previous studies where different inoculum ratios were used of toxigenic:non-toxigenic strains of *A. flavus*, which showed that 50:50 or 25:75 ratios, respectively, gave similar levels of AFB_1_ control as described previously (Medina et al., 2017a). The cob sections were point inoculated with 100 μl of the treatments after damage to a single kernel using a surface sterilized needle and incubated at 30°C for 10 days (Samsudin et al., 2017). In all cases, 3–4 replicates per treatment were used. Each environmental chamber included 250 ml of a sterile solution of glycerol/water to maintain the ERH at the same level as the cob ripening stage a_w_. The colony diameter was measured at the end of the incubation period. For *aflD* and *aflR* gene expression, contaminated kernels from the colonized area were carefully removed at random with a pair of forceps and immediately frozen in liquid N_2_ and kept at −80°C for subsequent RNA extraction and RT-qPCR as detailed previously (Al-Saad et al., 2016). The rest of the colonized cob was kept at −20°C for AFB_1_ extraction; clean-up was done using an immune-affinity column (IAC) and quantified by HPLC-FLD. The limit of detection was <1.0 ng/g.

### Post-harvest Studies With Non-GM and GM-Maize Cultivars

Two cultivars of non-GM maize and its isogenic GM lines were selected for the post-harvest biocontrol studies (Table 2). For these studies, batches of the maize grain were gamma-irradiated (12–15 kGy) in order to eliminate the natural contaminants but retain germinability of the kernels. The a_w_ of the maize treatments was modified to 0.98 and 0.95 with the addition of sterile water based on the moisture absorption curve for each cultivar. These were mixed thoroughly to ensure that the spore inoculum was well distributed throughout the maize grain. The number of spores added to each cultivar was calculated as 10 spores per gram of maize from a solution of 10^3^ spores/ml. After the addition of the water, the maize kernels were kept at 4°C for 24 h for full absorption and equilibration. The water availability was checked by measuring the a_w_ using the AquaLab® 4TE (Decagon, USA).

**Table 2:** Maize cultivars (non-GM and GM) selected for biocontrol *in situ* as substrate for *A. flavus* development.

Conventional cultivar (non-GM)	Isogenic line (GM)	Event name^1^	Inserted gene	Traits tolerance
M20-A78 CON	M20-A78 PW®	MON89034 + NK603 + TC1507	CP4 EPSPS	HT-Glyphosate
			PAT	HT-Glufosinate ammonium
			Cry2Ab2	IR-Lepidopteran
			Cry1F	
			Cry1A.105	
P30F53 CON	P30F53 H®	DAS1507 + T25	PAT	HT-Glufosinate ammonium
			BLA	Antibiotic resistance
			Cry1F	IR-Lepidopteran

*IR, insect resistance; HT, herbicide tolerance; PW®, PowerCore; H®, Pioneer Hi-Breed*.

1 Event name refers to the unique code to access the information about the trait at http://www.isaaa.org/gmapprovaldatabase/eventslist/default.asp

The non-toxigenic and toxigenic strains of *A. flavus* were incubated on MMA for 7 days at 30°C and then used to prepare the conidial spore inoculum. A final concentration of 10^3^ spores/ml was obtained. A ratio of 50:50 (toxigenic:non-toxigenic strains) was used. Previous showed that this as an effective inoculum load for biocontrol of AFB_1_ production (Mohale et al., 2013; Samsudin and Magan, 2016). The spore suspensions were mixed in a 50:50 ratio prior to inoculation of the maize kernels. The controls consisted of 100:0 and 0:100 ratios for the two control treatments.

A 10 g sample of maize grain were placed aseptically into glass culture vessels (Magenta™, Sigma, USA) with vented lids (10 mm with a polypropylene membrane 0.22 μm pore size) to allow gase exchange but keeping the environment inside the vessel sterile. These were inoculated with a 100 μl conidial mixture and shaken to distribute the conidia. The jars were placed in closed plastic environmental chambers that also contained a glycerol/water solution of the same a_w_ as the maize kernels to keep the ERH the same as the target treatments. The glycerol/water solution was renewed every 3 days. The environmental chambers were incubated at 30°C for 20 days. Samples were destructively sampled after 10 and 20 days storage for quantification of AFB_1_.

The samples for AFB_1_ quantification were oven dried at 65°C for 48 h to remove the water and stop fungal growth. These were then ground using a laboratory blender with a stainless steel blade (Waring, Stamford, USA). The effects on *aflD* and *aflR* gene expression were only examined after 10 days in all the treatments and replicates. The samples for gene expression were snap frozen in liquid nitrogen and stored at −80°C for subsequent RNA extraction. All studies were carried out with two non-GM and their isogenic GM cultivars. All experiments were carried out with at least three replicates per treatments and repeated once.

### Studies of Resilience of Non-toxigenic Strains of *A. flavus* When Controlling Aflatoxin B_1_ Contamination of Maize Cultivars Under Climate Change Conditions

#### Fungal Strains

Studies were carried out with two non-aflatoxigenic strains of *A. flavus* (AFL^−^:Mex02; AFL4^−^) isolated from Mexican and Brazilian maize, respectively, based on their ability to reduce AFB_1_ in previous *in vitro* and *in situ* studies (Rodríguez-Sixtos, 2017; Marcon Gasperini, 2019). The native toxigenic strains (AFL^+^MEX01; AFLb^+^; and the AFLe type strain) with known AFB_1_ production capacity were used as the toxigenic pathogen in these studies.

Pre-harvest studies involved the use of maize cobs at the R4 (dough) and R5 (dent) stages only. Post-harvest studies used the two non-GM and isogenic GM maize cultivars. The same modification procedures were used as detailed previously. A ratio of 50:50 conidia (toxigenic *A. flavus*: non-toxigenic *A. flavus*) were used. The controls consisted again of only the toxigenic or non-toxigenic conidial concentrations as detailed previously.

The post-harvest maize grain treatments were modified to 0.98 and 0.95 a_w_ with the addition of sterile water using the moisture adsorption curve of each cultivar to obtain the target treatment regimes. The maize treatments and replicates were inoculated with a conidial mixture of non-toxigenic and toxigenic strains as described previously (10^3^ conidia/ml and addition of the mixture resulting in approx. 10 conidia/g maize). The only exception was that for the cultivars AS 1555 CON and PRO®, and P2530 CON and Hx® were only conducted at 0.98 a_w_ because of a limited amount of maize grain of these cultivars available for the experiment.

For the CC study, the separate treatments were placed in plastic environmental chambers (Lock & Lock HPL890 16 L) containing a glycerol/water solution of the same a_w_ as the treatments as described previously. The environmental conditions were set to flush the CO_2_ treatments of 400 ppm (atmospheric CO_2_) and 1,000 ppm. The elevated CO_2_ content was achieved by using a gas cylinder containing a certified concentration of 1,000 ppm CO_2_/synthetic air at 200 bar prepared by the British Oxygen Company (Guildford, UK). The chambers were vented, and for the 1,000 ppm treatment flushed with CO_2_, every 12 h for the 20-day experimental period. The concentration of CO_2_ was regulated to 3 L/min (LPM) with a gas flow meter (Alicat Scientific, Arizona, USA) and flushed for 10–12 min, which corresponded to 2× the volume of each chamber. After flushing, the inlet and outlet valves of the chambers were immediately closed, and they were incubated at 30 or 35°C. The control chambers were flushed with air (400 ppm) and similarly incubated. At the end of the experiment, samples were destructively sampled for gene expression studies (10 days) and for AFB_1_ quantification (10, 20 days). The samples for toxin quantification were oven dried at 65°C for 48 h to remove the water and stop any fungal growth. The samples were ground using the laboratory blender as described previously.

### Gene Expression Studies

The gene expression studies were performed using the samples from the different maize ripening stages (pre-harvest studies) and those from the stored maize grain experiments after 10 days incubation. This time frame was chosen based on previous studies with both *A. flavus* and *A. parasiticus* that suggested gene expression of several of the toxin biosynthetic genes had optimal peaks of expression after 8–10 days growth (Schmidt-Heydt et al., 2008). The gene expression of the chosen genes was only performed for the interaction between toxigenic/non-toxigenic strains MEX01^+^:MEX02^−^ and AFLb^+^:AFL4^−^, respectively. The type strain AFLe^+^ (NRRL3357) treatments were not included because the AFB_1_ production was lower than the native Mexican or Brazilian toxigenic strains.

The treatments/replicates were stored at −80°C and transferred to reinforced 7 ml tubes designed for use in the Precellys 24® (Bertin, FR) homogenizer with three glass beads (6.5 mm). The tubes were kept in liquid N_2_ until use. The kernels were homogenized into a fine powder using a 6,500 rpm cycle for 30 s (2 × 15 s) and then immersing them in liquid N_2_ for 5 min and the cycle repeated. Approximately 50 mg of the powder was transferred to a 2 ml Eppendorf RNase/DNAse free to proceed with the extraction of the total RNA.

#### RNA Extraction

Total RNA isolation was carried out using the Spectrum™ Plant Total RNA Kit (Sigma-Aldrich Co, USA) according to the manufacturer instructions. The observations for samples with high amounts of starch were taken into account. For this reason, samples were incubated at room temperature, and 1 ml of lysis buffer added to the 50 mg of powder. RNA samples were treated with RNase-Free DNAse set (Qiagen, Hilden, Germany). The purity and concentrations of RNA were examined by measuring the absorbance of 2 μl of sample Genova-Nano spectrophotometer (JenWay, Staffordshire, UK). Samples were considered to be of good purity when the ratio A_260_/A_280_ was ≥2.0. The RNA integrity was verified using Experion™ RNA StdSens in an Experion™ automated electrophoresis system (Bio-Rad, California, USA) or by gel electrophoresis, and an RQI >6 was used as a threshold for integrity. The samples were kept at −80°C until use.

#### Relative Gene Expression Using Quantitative Polymerase Chain Reactions

Real-time quantitative PCR (RT-qPCR) assays were used to amplify the structural *alfD* and the regulatory gene *afl*R of the aflatoxin biosynthetic pathway as target genes (Table 3). The β-tubulin gene was used as the control gene. The *afl*D qPCR was previously optimized by Abdel-Hadi et al. (2010), whereas *afl*R was optimized by Medina et al. (2015) following the same method as that for the *afl*D gene.

**Table 3:** Nucleotide sequences of primers for RT-qPCR assays designed on the basis of the *aflD*, *aflR,* and β-tubulin genes.

Primer pairs	Gene	Nucleotide sequences (5′–3″)	Position
*nor*Taq – 1	*aflD*	GTCCAAGCAACAGGCCAAGT	516^a^
*nor*Taq – 2		TCGTGCATGTTGGTGATGGT	562^a^
*nor-*Probe		[FAM]TGTCTTGATCGCGCCCG[BHQ2]	537^a^
*afl*RTaq – 1	*aflR*	TCGTCCTTATCGTTCTCAAGG	1,646^b^
*afl*RTaq – 2		ACTGTTGCTACAGCTGCCACT	1,735^b^
*afl*R-Probe		[FAM]AGCAGGCACCCAGTGTACCTCAAC[BHQ2]	1,689^b^
benTaq – 1	β-tubulin	CTTGTTGACCAGGTTGTCGAT	65^c^
benTaq – 2		GTCGCAGCCCTCAGCCT	99^c^
ben-Probe		[CY5]CGATGTTGTCCGTCGCGAGGCT[BHQ2]	82^c^

a *Positions are in accordance with the published sequence of the *aflD* gene of *Aspergillus flavus* (GenBank accession no. XM_002379908.1)*;

b *Positions are in accordance with the published sequences of *aflR* gene of *Aspergillus flavus* (GenBank accession no. AF441435.2)*;

c *Positions are in accordance with the published sequences of β-tubulin (benA56) gene of *Aspergillus flavus* (GenBank accession no. AF036803.1)*.

Two RT-qPCR assays were carried out, one optimized to amplify the target *afl*D and the housekeeping β-tubulin genes, and the other one to quantify the *afl*R gene expression and for the β-tubulin gene. The qPCR reactions were prepared in triplicate for each biological replicate (*n* = 9). The TaqMan system with different primers and probes was used in all cases. Both reaction mixtures consisted of 6.25 μlm Premix Ex Taq™ (Takara Bio Inc., Otsu, Japan), 830 nM of each primer, 330 nM of each probe, and 1.5 μl of cDNA template in a final volume of 12.5 μl. The optimal thermal cycling conditions included an initial step of 10 min at 95°C and all 50 cycles at 95°C for 15 s, 55°C for 20 s, and 72°C for 30 s. The assays were carried out using a CFX96 Touch™ Real-Time PCR detection system (Bio-Rad, CA, USA).

#### Relative Quantification of the Expression

Relative quantification of *afl*D and *afl*R genes was performed using the housekeeping gene β-tubulin (ben) as an endogenous control to normalize the quantification of the target in the relative quantification assays and used for all treatments. Quantification cycle (Cq) determinations were automatically performed by the instrument using the default parameters, and the expression ratio was calculated using the 2^−∆∆Ct^ method as proposed by Livak and Schmittgen (2001). The control of each condition corresponded to AFLb^+^ without the presence of the BCA.

### Mycotoxin Quantification

For the extraction of AFB_1_, a 2 g sample was ground and transferred to a glass vial and mixed with 8 ml of extraction solution (methanol:water; 80:20 v/v). The samples were agitated with a magnetic stirrer for 1 h at room temperature and then centrifuged to allow phase separation. The liquid phase was transferred to a new 15 ml polypropylene tube and 100 μl of the extract mixed with 900 μl of mobile phase (methanol:acetonitrile:water 30:15:60 v/v/v) in a 2 ml Eppendorf tube and vortexed and filtered using a nylon filter (13 mm × 0.22 μm) directly into an amber silanized vial. The samples were injected into the HPLC system, and samples below the limit of detection (<1.0 ng/g) were cleaned-up and concentrated using an IAC as described previously and then reinjected.

#### IAC Analysis

The extract of the samples was diluted 2:20 in 1× PBS (phosphate buffered saline, Fisher Scientific, USA). The pH of the extract was checked to ensure that this was not lower than 7.0 to ensure good performance of the IACs (AflaStar™ R, RomerLabs, Austria). The IACs were brought to room temperature prior to use and were attached to a SPE vacuum manifold (Phenomenex, CA, USA). Above each IAC, a 25 ml reservoir was used to hold the sample extract. Following the manufacturers’ instructions, the buffer in the IAC was removed, and the sample extract passed through the column at a speed of 1 to 3 ml/min. This was followed by 20 ml of 1× PBS for the clean-up (10 ml was added in the reservoir and 10 ml added directly into the IAC). The last step was the elution with 1.5 ml of methanol. For best recovery, the elution was performed by adding 3 × 500 μl of methanol. The eluted samples were evaporated to dryness using a vacuum evaporator (miVac Quattro Concentrator – Genevac, Leicestershire, UK) at 45°C for 3 h. The dried extract was re-suspended in 500 μl mobile phase, transferred to an amber silanized vial, and injected into the HPLC for AFB_1_ quantification.

#### HPLC Analysis

The quantification of AFB_1_ in the maize grain was done by reverse-phase HPLC. The HPLC system used was an Agilent 1,200 series (Agilent, Santa Clara, USA) with a fluorescence detector (λ_exc_ 360 nm; λ_em_ 440 nm) and post-column derivatization with a UVE photochemical reactor with UV-Light (LCTech GmbH, Germany). A C_18_ column (Agilent Zorbax® Eclipse Plus, 2.1 × 100 mm, 3.5 μm particle size) preceded by a Phenomenex® Gemini C18 guard column cartridge 3 mm × 3 μm (Phenomenex, CA, USA) was used for separation. Followed by isocratic elution with methanol:water:acetonitrile (30:60:15, v/v/v) and a mobile phase flow rate of 1.0 ml/min. The injection volume was 5–50 μl according to each set of samples. A set of standards was injected (0.05–4 ng of a mixture of aflatoxins per injection), and standard curves were generated by plotting the peak areas against the amounts of each aflatoxin. The recovery of the extraction method for AFB_1_ in maize was 80%.

### Statistical Analyses

The data from pre-harvest cobs of different ripening stages and the *in situ* post-harvest experiments (gene expression data, AFB_1_) were analyzed using the Shapiro-Wilk tests to determine normality and Levene’s test to assess variance homogeneity. However, the data violated the two assumptions for ANOVA even after transformations and therefore non-parametric tests (Kruskal-Wallis/Wilcoxon; *p* = 0.05) were used for analyses (Chan and Walmsley, 1997). Where there was significance after the Kruskal-Wallis test, median comparisons for each pair were made using the Wilcoxon-Each Pair test (*p* = 0.05). The correlation of relative gene expression × AFB_1_ production was checked using non-parametric Spearman’s (*ρ*) rank correlation coefficient for each a_w_ level. The statistical package JMP®14 (SAS Institute Inc., 2018, Cary, NC, USA) was used to perform the analyses.

The data sets for the effect of CC scenarios on AFB_1_ production satisfied the requirements for ANOVA after transformation to the cube root. Tests were thus performed comparing the interactions of temperature × CO_2_ × a_w_ for each cultivar of maize. The relative gene expression for this study violated the two assumptions for ANOVA, and the differences were compared using non-parametric tests (Kruskal-Wallis/Wilcoxon; *p* = 0.05). The calibrant (control sample) for the biocontrol experiment was the toxigenic pathogen strain (AFLb^+^) in the same conditions as the non-toxigenic strain was applied. For the effects of CC, the control sample refers to normal environmental conditions (30°C, 400 ppm CO_2_) for each cultivar. The statistical package JMP®14 (SAS Institute Inc., 2018, Cary, NC, USA) was used to perform the analyses.

## Results

### Effect of Maize Cob Ripening Stage and Temperature on Colonization and Aflatoxin B_1_ Contamination

**Figure 1:**
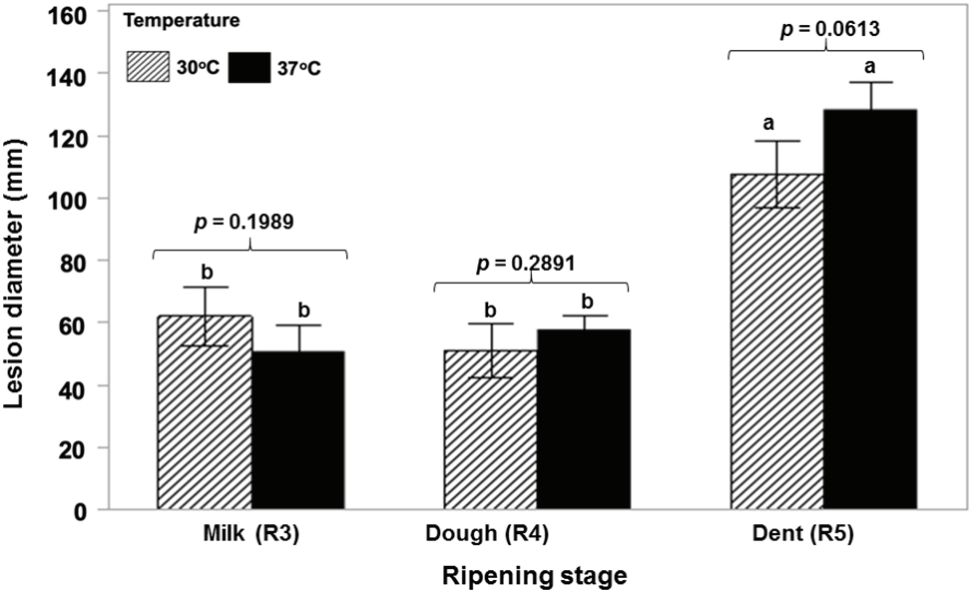
Relative colonization rates of maize cobs of different ripening ages (R3, R4, R5) by a toxigenic strains of *A. flavus* (NRRL 3375) when point inoculated and incubated at the water activity levels of the different growth stages at 30 and 37°C. Statistical test performed after lesion diameter (mm) data was transformed to Log(x + 1) to achieve normality fit (Shapiro Wilk *p* ≥ 0.05). The values of *p* indicate no difference between temperatures. Treatments with the same letters indicate no differences in the ripening stages based on the Tukey’s HSD test (*p* ≥ 0.05).

**Figure 2:**
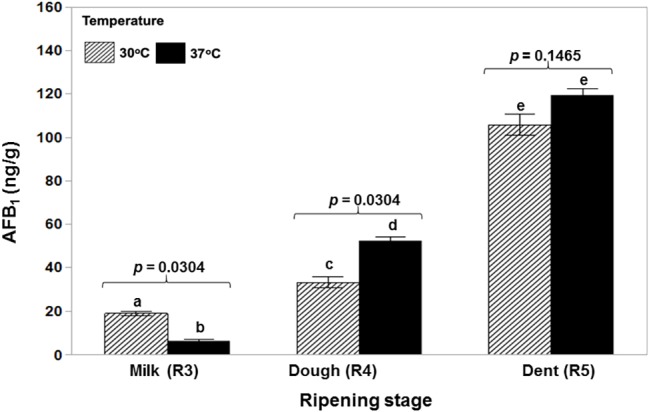
Relative contamination of maize cobs of different ripening ages (R3, R4, R5) with aflatoxin B_1_ when incubated at the actual water activity levels of the maize kernels at the different growth stages at 30 and 37°C. The toxigenic strain NRRL 3375 was used. The values of *p* indicate no evidence of difference between temperatures. Ripening stage treatments with the same letters were not significantly different using the Wilcoxon for each pair test (*p* ≥ 0.05). Overall analyses was done using the Non-Parametric data – Wilcoxon Test. Ripening stages: R3 × R4 × R5, *p* = 0.0009; Temperatures: 30 × 37°C, *p* = 0.7728.

Figure 1 shows that a toxigenic strain of *A. flavus* is able to colonize ripening maize cobs of different ages at both 30 and 37°C in a relatively similar manner with no significant difference between cob ripening stage and temperature. AFB_1_ production was significantly lower initially at the R3 (milk) stage and 37°C and then significantly higher at the R4 (dough) stage and the same temperature. There was no difference at the R5 (dent) stage (Figure 2). Statistically, there was a significant overall effect of ripening stage but not of temperature.

### Relative Control of AFB_1_ Contamination in Ripening Ages of Maize Cobs Using a Non-toxigenic *A. flavus* Strain

**Figure 3:**
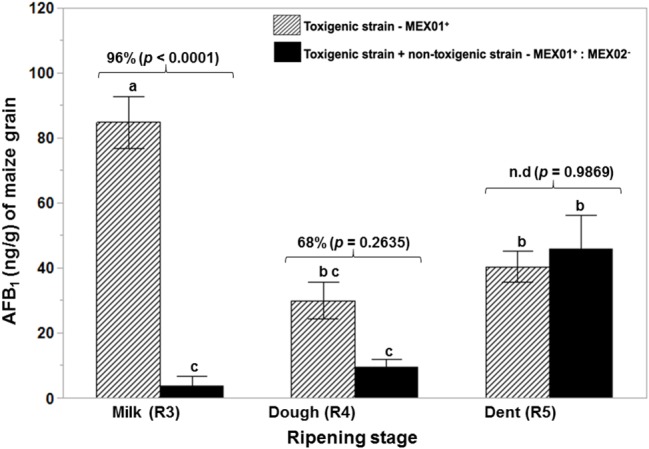
Effect of a non-toxigenic strain (AFL-MEX02) of *A. flavus* when co-inoculated with a wild type toxigenic strain (AFL + MEX01) on aflatoxin B_1_ production when applied as a mixed inoculum (50:50) to maize cobs of different ripening ages (R3, R4, R5) after 10 days at 30°C. The percentage values above the bars show relative reduction of AFB_1_, and the values of *p* (<0.05) indicate evidence of difference from the control (toxigenic strain). Treatments with the same letters show no differences in the ripening stages using the Tukey’s HSD test at 5% significance. n.d. – AFB_1_ reduction not detected.

**Plate 1:**
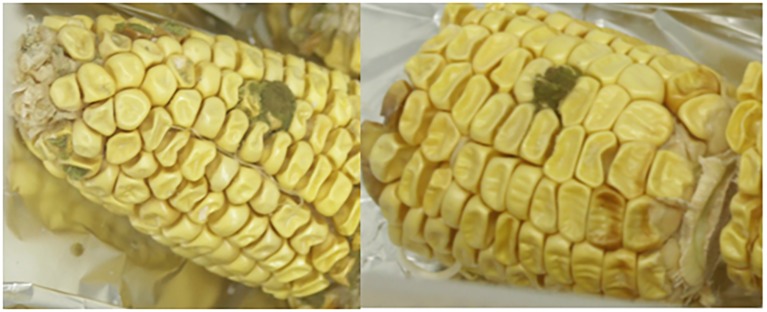
Example of colonization of maize cob sections by mixtures of 50:50 inoculum of the toxigenic and non-toxigenic strains (AFL + MEX01:AFL-MEX02), respectively, at the R4 stage.

**Figure 4:**
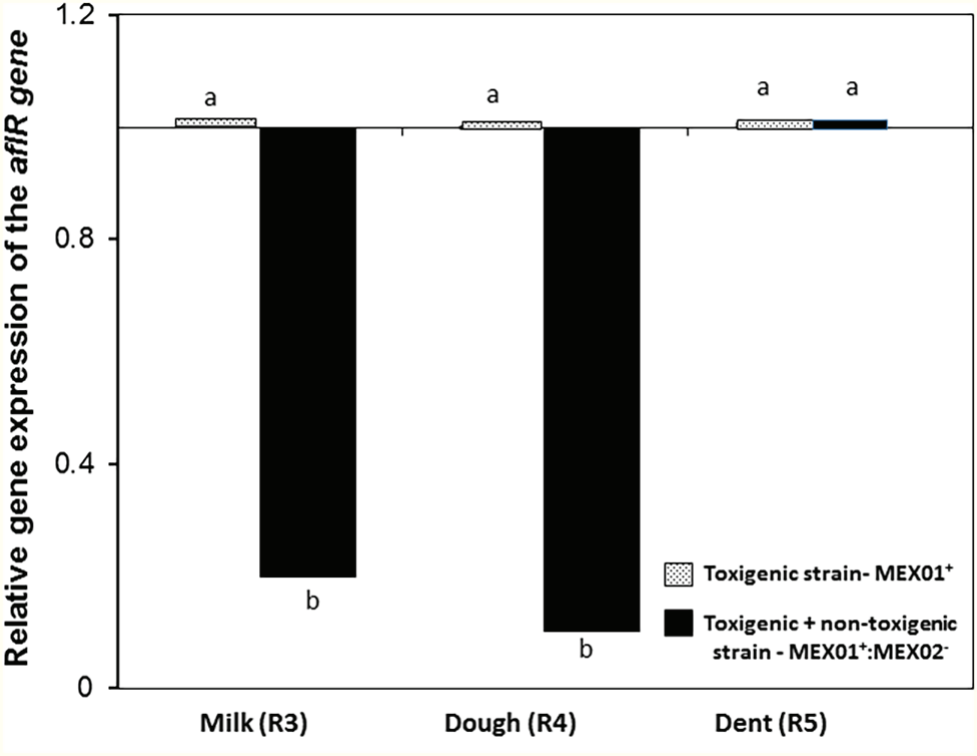
Relative gene expression of the regulatory *aflR* gene in maize cobs of different ripening stages when co-inoculated with the non-toxigenic and toxigenic strains of *A. flavus* (AFL-MEX02+ AFL + MEX01) after 10 days incubation at 30°C. Treatments with different letters are significantly different (*p* = 0.05).

Figure 3 shows the effect of the non-toxigenic strain (AFL^−^:MEX02) on control of AFB_1_ production by the toxigenic strain (AFL^+^MEX01) on maize cobs of different ripening stages. This shows that the level of control of AFB_1_ was maximum at the R3 (milk) and R4 (dough) stages (*p* = 0.05). At the R5 (dent) stage, there was no control of toxin production. Plate 1 shows an example of the colonization of maize cobs segments at the R5 stage. This was confirmed by measurement of the relative expression of the regulatory *aflR* gene, which was downregulated at the R3 and R4 stages in the maize cobs, while in the R5 stage, there was no difference from the toxigenic control gene expression levels (Figure 4).

### Post-harvest Control of Aflatoxin Under Different Water Availability Conditions in Non-GM and GM Maize Grain

**Figure 5:**
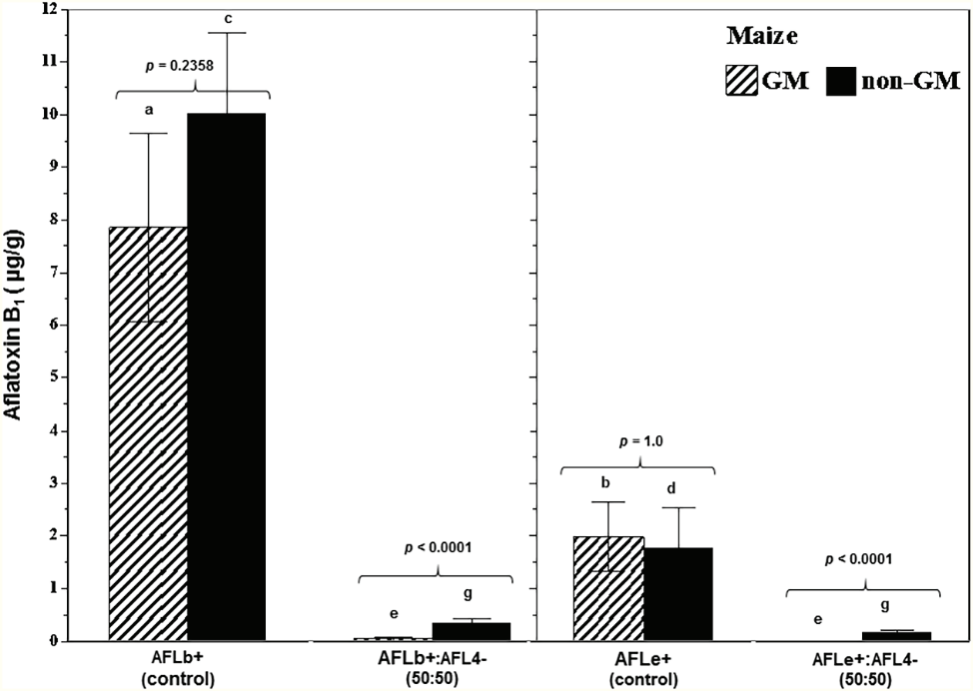
Overall aflatoxin B_1_ contamination of post-harvest stored maize grain treated with a mixture of a non-toxigenic and a toxigenic strain of *A. flavus* in non-GM and GM maize cultivars taking into account the parameters of water availability (0.98, 0.95), time of incubation (10, 20 days), and type of maize cultivar (non-GM vs. GM). The grain was inoculated with two different Brazilian or NRRL type toxigenic strains (AFL^+^ or AFLe^+^) and the non-toxigenic Brazilian strain AFL4^−^ in a 50:50 inoculum ratio. Different letters indicate a significant difference (*p* < 0.05) between the strains of the same maize type. The values of *p* show differences in overall AFB_1_ content comparing GM and non-GM maize at 5% significance. Bars represent mean ± SE.

**Figure 6:**
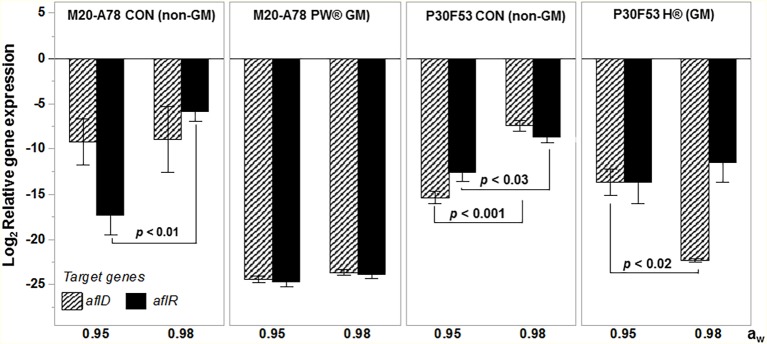
Effect of biocontrol using the Brazilian AFL4^−^:AFLb^+^ mixed non-toxigenic and toxigenic strain 50:50 ratio inoculum on the relative gene expression of the structural *aflD* and regulatory *aflR* genes on one of the non-GM and isogenic-GM cultivars stored post-harvest at both 0.98 and 0.95 water activity. The values of *p* show differences in overall AFB_1_ content comparing GM and non-GM maize at 5% significance. Bars represent mean ± SE.

Figure 5 summarizes the effect of a non-toxigenic strain (AFL4^−^) on AFB_1_ control in maize grain contaminated with two different toxigenic *A. flavus* (AFLb^+^; AFLe^+^) strains on AFB_1_ control in GM and non-GM cultivars (mean of two cvs of each). This showed that the toxigenic AFLb^+^ strain produced significantly more AFB_1_ than the AFLe^+^ (NRRL3375) strain. Overall, there was better control of AFB_1_ in the GM-maize cultivars than the non-GM ones. Although in both GM and non-GM cultivars, >90% control of AFB_1_ contamination of the maize grain was achieved using the initial inoculum ratio of 50:50 of non-toxigenic:toxigenic conidial inoculum. These studies were carried out at 30°C and showed that efficacy was consistent across non-GM and GM cultivars. Figure 6 compares the relative expression of both the *aflD* and *aflR* genes in one of the non-GM and GM cultivars at 0.98 and 0.95 a_w_. The efficacy of the non-toxigenic strain on the toxigenic strains was supported by the effects on the structural and regulatory genes examined. These were significantly downregulated in both non-GM and GM cultivars, although this was more pronounced in the latter one.

### Resilience of Non-toxigenic Strains of *A. flavus* for Control of Aflatoxin B_1_ Under Three-Way Interacting Climate Change Abiotic Conditions in Non-GM and GM Strains

Two sets of studies were carried out. Pre-harvest studies with a 50:50 mixed conidial inoculum of the non-toxigenic:toxigenic strains were used in maize cobs at the R4 and R5 stages and incubated at 30 or 35°C and exposed to elevated CO_2_ for 10 days. This showed that there were no statistically significant differences in AFB_1_ contamination between the control and the elevated CO_2_ treatments (*p* = 0.05; means of R4: 545 vs. 390; R5 = 1,125 vs. 780 ng/g AFB_1_ in maize, respectively). The relative expression of the *aflD* and *aflR* genes showed that there was an inhibition of the former structural gene expression but not of the latter regulatory gene (data not shown).

**Figure 7:**
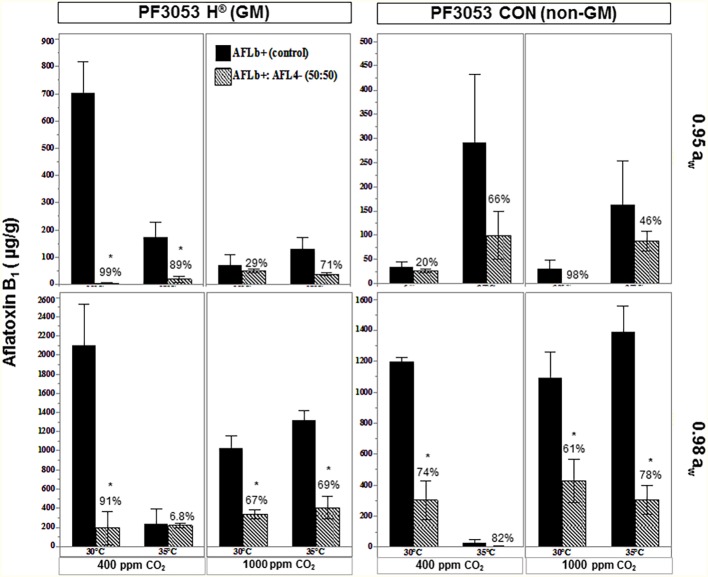
Effect of temperature (30, 35°C), CO_2_ (400, 1,000 ppm), and water activity (a_w_, 0.98, 0.95) on aflatoxin B_1_ contamination by the Brazilian toxigenic strain AFLb^+^ (control) and biocontrol mixture of AFLb^+^:AFL4^−^ conidial ratio 50:50 pathogen:antagonist in conventional (P30F53 CON) and isogenic GM (P30F53 H®) stored maize kernels. Values above bars represent relative control (%) of aflatoxin B_1_; ^*^represent significant reduction (*p* < 0.05) of aflatoxin B_1_ from the toxigenic control (AFLb^+^).

Exposure to interacting climate change abiotic factors in post-harvest storage studies was carried out with the two non-GM and isogenic GM cultivars. Figure 7 compares the relative effect of the biocontrol strain AFL4^−^ when co-inoculated with the toxigenic strain AFLb^+^ in one of the non-GM and GM cultivars when carried out under existing CO_2_ conditions (400 ppm) at two a_w_ stress levels and comparisons with exposure to 1,000 ppm CO_2_. There was higher AFB_1_ production at 0.98 a_w_ than at 0.95 a_w_. In addition, in the non-GM cultivar, more AFB_1_ was produced by the toxigenic strain. Overall AFB_1_ control was more effective at 0.98 a_w_ than at 0.95 a_w_ in both existing and future CO_2_ scenarios. For both GM and non-GM maize cultivars, the control of AFB_1_ was similar, suggesting relative resilience of this non-toxigenic strain.

**Figure 8:**
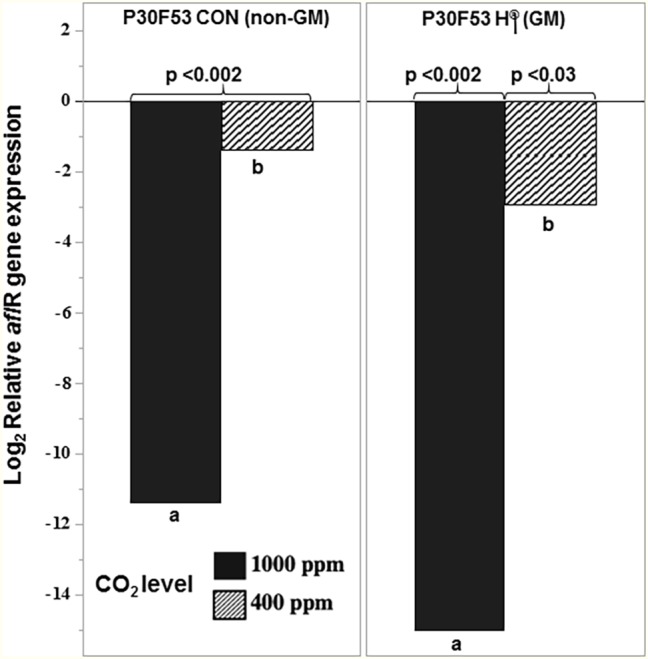
Effect of the non-toxigenic *A. flavus* strain AFL4^−^ when applied as a conidial inoculum in the ratio of 50:50 with the toxigenic strain (AFL4^−^:AFLb^+^) on relative expression of the regulatory gene *aflR* in the conventional (P30F53 CON) and GM (P30F53 H®) stored maize grain at 0.98 a_w_ after 10 days at 35°C in 400 and 1,000 ppm CO_2_ levels. The expression was normalized for the toxigenic control treatment (AFLb^+^) in each condition. The values of *p* represent significant differences from the control sample; different letters indicate effects of the CO_2_ treatment levels (*p* < 0.05).

Figure 8 compares the relative expression of the regulatory *aflR* gene at 35°C and 0.98 a_w_ in the different CO_2_ treatments (400 and 1,000 ppm CO_2_) with the control at 30°C/400 ppm CO_2_ in both non-GM and GM cultivars. This shows that the *aflR* gene expression was significantly affected at 35°C when comparisons were made between existing (400 ppm) and future (1,000 ppm) CO_2_ treatments. The relative expression values were relative to those at 400 ppm CO_2_ and 30°C (existing conditions).

## Discussion

This study has examined the pre-harvest and post-harvest resilience of non-toxigenic strains of *A. flavus* for control of AFB_1_ contamination of maize cultivars, including non-GM and isogenic non-GM with herbicide/pesticide resistant traits for the first time. The ability of the toxigenic strains of *A. flavus* to colonize ripening maize cobs at both 30 and 37°C suggests that it is important to screen non-toxigenic biocontrol strains for resilience to fluxes in temperature and the ability to tolerate a range of water availability conditions to ensure that competitiveness both pre- and post-harvest can be maintained. The fact that AFB_1_ control was more effective at the milky ripe (R3) and dough stages (R4) of maize cobs pre-harvest suggests that the window for control based on the a_w_ range at these stages may be an important consideration for application of the biocontrol strain. Recently, Giorni et al. (2019) showed that relative infection of ripening maize cobs by *A. flavus*, *Fusarium verticillioides* and *F. graminearum* influenced the colonization and the mixture of mycotoxins contaminating the maize at harvest. Thus, the presence of *A. flavus* impacted on the contamination of the maize grain with other mycotoxins such as fumonisin B_1_ (FB_1_) and deoxynivalenol, perhaps because of the its wider range of temperature and water availability during silking.

The biocontrol of AFB_1_ contamination at different ripening stages is important. They represent different a_w_ levels as well as different nutritional compositions during the silking process. However, since ripening stage did not affect the ability of the toxigenic strain to colonize the cobs, this suggests that toxigenic *A. flavus* strains are able to colonize maize cobs rapidly during silking, if entry points are available for infection. Thus, the resilience of the non-toxigenic biocontrol strains is critical to facilitate niche exclusion or effectively outcompete the toxigenic strains either in soil or in the later silking process (Medina et al., 2017b). Previously, it has been suggested that *A. flavus* is adapted to the ripening stages of maize, expressing specific genes to utilize the available carbon sources (CS; Reese et al., 2011; Dolezal et al., 2014). The non-toxigenic strain was effective in controlling AFB_1_ production at the milky ripe and dough stages. However, at the dent stage, it was less effective. This was supported by the effects on the gene expression of one of the key regulatory genes (*aflR*) involved in secondary metabolite production. Previously, Verheecke et al. (2015) studied the efficacy of *Streptomyces* strains against toxigenic *A. flavus* strains on synthetic media. They examined five different biosynthetic genes involved in aflatoxin production. In their study, the *aflR* expression was decreased by a *Streptomyces* strain, but *aflD* expression was unaffected by all the biocontrol strains examined. However, these studies did not include the impact of water availability, which may have affected the relative control achieved. In the study by Al-Saad et al. (2016), the efficacy of bacterial biocontrol strains showed significant efficacy with a decrease in the *aflD* and *aflR* relative expression, although this was not always translated into effective phenotypic toxin control.

The non-toxigenic strain AFL4^−^ was able to significantly reduce the AFB_1_ content *in situ* when paired with two different toxigenic strains, AFLb^+^ and the type strain NRRL3357 (AFLe^+^) in a 50:50 inoculum ratio. The relative AFB_1_ control ranged from 58 to 100% under different a_w_ (0.98 and 0.95) conditions for up to 20 days storage. Furthermore, the present study has shown for the first time the comparison between impacts of such biocontrol in the reduction of AFB_1_ in conventional (non-GM) and isogenic GM maize cultivars. The overall AFB_1_ control indicated that the non-toxigenic strains applied were less resilient in non-GM stored maize cultivars, with the final control achieved being lower than that in the isogenic GM cultivars.

The question is whether this is due to differences in the biochemical composition of non-GM and GM cultivars due to the manipulation of the genes, e.g., the presence of the Cry1Ab gene. There are few studies, which have examined this. There is a study, which has suggested that Cry1Ab protein in maize residues had no direct effect on *F. graminearum* and *Trichoderma atroviride*. However, some corresponding BT/non-BT maize hybrids differed more in composition than that due to the Cry gene alone. However, this may affect the saprophytic growth of such fungi on crop residues (Naef et al., 2006). Changes in free fatty acids (FFA) in maize are an indicator of fungal infections and a deterioration in quality. Thus, fatty acids (FAs) in the maize could play a role in pathogen susceptibility and seed colonization (Dall’Asta et al., 2012). FFAs, especially of linoleic acid levels, partly regulate development, colonization, and mycotoxin production by *Aspergillus* spp. (Scarpari et al., 2014). Thus, changes in nutritional or environmental factors or both may also influence secondary metabolite production (Magan and Aldred, 2007).

The efficacy of the non-toxigenic strains in controlling AFB_1_ production was supported by the gene expression of the target structural and regulatory genes (*afl*D, *afl*R). The results showed that competition in the maize grain niche inhibited the relative expression of both these genes. The correlation with phenotypic AFB_1_ production showed that the lowered expression of these genes resulted in less toxin after 10 days storage. Previously, Al-Saad et al. (2016) when screening bacterial antagonists as biocontrol agents of toxigenic *A. flavus* strains sometimes found that while relative inhibition of *afl*D and *afl*R expression was evident, sometimes there was concomitant stimulation of AFB_1_ production. Thus, the resilience of such potential biocontrol strains may well be influenced by water and temperature stress limitations, as well as inoculum ratio × nutritional parameters to avoid such effects. Recently, Venkatesh and Keller (2019) suggested that there are complex interactions between bacteria and mycotoxigenic fungi and focused on key ecological factors being light, nutrients, and pH. Two probably more important abiotic factors are water availability and temperature. Bacteria require almost freely available water (>0.98–0.99 a_w_) for growth and have significantly less resilience to water stress than many mycotoxigenic fungi, which are either xerotolerant or xerophilic, the only exceptions being some of the toxigenic Fusaria. However, stored cereals are seldom harvested or stored under wet conditions, and thus, the interactions with bacteria may in fact be relatively limited, although pre-harvest interactions certainly must occur. Thus, the competitive exclusion of toxigenic strains of *A. flavus* is more likely to be successful with non-toxigenic strains or relatively xerotolerant species that may have the temperature and water stress tolerance to compete effectively. Indeed, Kohl et al. (2019) suggested that biocontrol agents use a cascade of mixed mechanisms of action to control plant pathogens, and this needs to be borne in mind when developing biocontrol strategies, including for toxigenic fungal pathogens.

It was noted that the relative gene expression of biosynthetic genes of the toxigenic *A. flavus* strains in non-GM and isogenic GM-maize was similar for the *afl*D and *afl*R at 30°C after 10 days storage. However, the phenotypic AFB_1_ production was different in the two types of cultivars. For example, in the conventional M20-A78 CON cultivar, the relative reduction in AFB_1_ was lower, and this was supported by the less pronounced reduction in the gene expression of the two genes examined. Furthermore, the a_w_ level also had an effect on the relative gene expression when the non-toxigenic strain was applied, under the same storage conditions. Previously, Abdel-Hadi et al. (2010) reported that under water stress levels (e.g., 0.90 a_w_), the *afl*D expression can be increased, although it is optimally expressed at 0.98 a_w_ on a conducive medium.

It has been shown previously that the ratio of the two regulatory genes *aflR* and *aflS* changes with a_w_ × temperature conditions (Abdel-Hadi et al., 2012). The *afl*R gene encodes for a positive regulator (AFLR), which activates the pathway gene transcription (Chang et al., 1995), whereas the *afl*S (=*afl*J) gene encodes for a protein factor (AFLS), which is involved in the regulation of transcription. These key genes are adjacent to and associated with the expression of a number of structural genes, e.g., *afl*C, *aflD* (*nor*-1), *afl*M (*ver*-1), and *afl*P (*omt*A; Chang and Hua, 2007). The capacity for disrupting the functioning of either or both of these two genes (*afl*S, *afl*R) perhaps by competition from non-toxigenic strains can reduce or completely inhibit aflatoxin production (Meyers et al., 1998; Yu et al., 2004).

This study has explored the effects of elevated CO_2_ and temperature on potential for control by non-toxigenic fungi, both pre- and post-harvest with both non-GM and GM cultivars. Pre-harvest results suggest that the non-toxigenic strain used (MEX02^−^) was not as resilient under elevated climate change interacting factors with no effective control of AFB_1_ production in maize cobs of the dent stage. The post-harvest studies were able to examine the relative efficacy of the non-toxigenic strain (AFL04^−^) on control of AFB_1_ in related non-GM and isogenic GM cultivars for the first time. The effect of increasing CO_2_ at 30 and 35°C varied depending on the type of maize used (conventional or GM). This was the first attempt to analyze whether using these two types of cultivars under climate-related abiotic factors can cause differences in AFB_1_ production and the level of control achieved when using non-toxigenic biocontrol strains.

The action of the non-toxigenic *A. flavus* strain (AFL4^−^) as a biocontrol agent was significant in the elevated CO_2_ treatments, although the overall efficacy was lower than in non-climate change abiotic conditions. The use of the GM cultivar (P30F53 H®) showed better results in terms of relative biocontrol under abiotic stress (0.95 a_w_) and increased CO_2_.

There are limited previous studies to examine the effect of three-way interacting abiotic factors of elevated temperature × elevated CO_2_ × drought stress effects on biocontrol of mycotoxin production (Verheecke-Vaessen et al., 2019). Previously, three-way interacting climate change abiotic factors were shown to stimulated AFB_1_ production by the type strain of *A. flavus* (NRRL strain) both *in vitro* and in stored maize grain under increased temperature (30 vs. 34–37°C), 350 vs. 650/1,000 ppm, CO_2_, and different a_w_ stress levels (Medina et al., 2015, 2017b). While growth of *A. flavus* was relatively unaffected, expression of key genes such as the *aflR* and *aflD* was significantly increased and translated into a stimulation of AFB_1_ production. Indeed, Vaughan et al. (2014) demonstrated that elevated CO_2_ (800 μmol CO_2_ mol^−1^ air) enhanced maize susceptibility to *F. verticillioides* infection, but the increase in fungal biomass did not result in higher FB_1_ toxin levels. Although subsequent studies suggested that there was an interaction between drought stress and elevated CO_2_, which increased FB_1_ production (Vaughan et al., 2016).

More recently, Gilbert et al. (2018) using RNA-Sequencing demonstrated that AFB_1_ production in stored maize grain was altered by a_w_ × temperature × elevated CO_2_. Also, several genes involved in the biosynthesis of secondary metabolites exhibit different responses to a_w_ or temperature stress depending on the atmospheric CO_2_ content. At 37°C and 1,000 ppm CO_2_, the transcription factor *afl*R was decreased. After 10 days incubation, the expression of biosynthetic genes in maize stored at 30°C generally decreased. However, the effects of high CO_2_ (1,000 ppm) and water stress (0.91 a_w_) showed decreased values, possibly in response to elevated AFB_1_ levels (Gilbert et al., 2018).

Other imposed chemical stresses may also result in physiological impacts on toxigenic fungi such as *A. flavus*. Recent studies by Hanano et al. (2019) have shown that the most toxic congener of dioxin, the 2,3,7,8-tetrachlorinated dibenzo-p-dioxin, reduced growth but stimulated both conidial sporulation and AFB_1_ production supported by levels of biosynthetic gene expression. Indeed, such exposure to chemical stress was shown to increase the production of superoxide dismutase and catalase. Of particular interest was the activity of caleosin/peroxygenase enzyme, which was activated in the presence of such recalcitrant compounds. This suggests that exposure to elevated levels of atmospheric particulates and gaseous stresses such as CO_2_ and interaction with elevated temperatures may impact on physiological functioning of *A. flavus* and influence the toxin contamination levels.

## Conclusions

This study has shown that the relationship between pre-harvest ripening stage of maize cobs and their inherent water availability will influence both colonization and AFB_1_ production by toxigenic *A. flavus* strains and also influence the potential for effective control of toxin contamination. Thus, by using a 50:50 ratio of BCA:pathogen composition, the efficacy of the non-toxigenic strain was more effective at the R3 and R4 ripening stages, supported by the downregulation of the two toxin genes (*aflD, aflR*) relative expression, accompanied by a significant reduction in AFB_1_ contamination. At the dent stage, perhaps a higher inoculum of the non-toxigenic strain is necessary for effective control.

Post-harvest, biocontrol of AFB_1_ production in non-GM and GM cultivars was affected by interacting variables: type of cultivar, *T* °C, CO_2_ levels, and water availability conditions. These interactions may also significantly affect BCA resilience and relative action. This study suggests that in GM cultivars, the relative control was slightly more effective than in the equivalent isogenic non-GM maize cultivars post-harvest. The resilience of the non-toxigenic strains appeared to vary between these. Overall, it is very important to include resilience to climate-related abiotic factors to ensure that the identified strains of non-toxigenic strains and indeed other biocontrol candidates have the necessary ecological competence to compete effectively and reduce toxin contamination, whether the approach is for controlling pre- or post-harvest. Formulation approaches for such biocontrol agents may also play an important role in conserving resilience under a range of interacting abiotic conditions in the maize agroecosystem.

## Data Availability Statement

All the data sets are deposited with Cranfield University *via* the senior author and can be openly accessed *via* him.

## Author Contributions

AG and AR-S carried out the research work. EG-C and CV-V assisted with molecular work and quantification of toxins. AM and NM supervised the research and drafted the manuscript.

### Conflict of Interest

The authors declare that the research was conducted in the absence of any commercial or financial relationships that could be construed as a potential conflict of interest.
